# When Air Strikes: A Pregnant Patient’s Surprising Encounter With Pneumoperitoneum

**DOI:** 10.7759/cureus.78517

**Published:** 2025-02-04

**Authors:** Clement Rajakumar, Brittany Al-atrache, Steven Tizio

**Affiliations:** 1 General Surgery, Jersey Shore University Medical Center, Neptune, USA

**Keywords:** abdominal distention, exploratory laparotomy, free air, idiopathic, pneumoperitoneum, pregnancy, substance use disorder

## Abstract

Pneumoperitoneum, characterized by free air in the peritoneal cavity, typically signals significant intra-abdominal pathology, often due to perforated hollow viscera requiring urgent surgical intervention. However, spontaneous idiopathic pneumoperitoneum, particularly in pregnant patients, is rare and poorly understood. This case report details a 41-year-old female, 24 weeks pregnant status post two rounds of cardiopulmonary resuscitation (CPR), who presented unresponsive after a suspected overdose. Initial assessments revealed abdominal distention and a CT scan indicated substantial intra-abdominal free air. An exploratory laparotomy was performed, which, surprisingly, showed no evidence of perforation or ischemia in any abdominal organs. Following surgery, the patient remained stable, was extubated on postoperative day one, and progressed to a regular diet, although she declined recommended psychiatric care for substance use disorder. This case highlights the diagnostic complexities of pneumoperitoneum in pregnancy, suggesting that factors such as trauma, physiological changes, and substance-related gastrointestinal dysmotility may contribute to this condition. The absence of identifiable perforation emphasizes the potential for benign causes, necessitating a multidisciplinary approach to effectively address the needs of pregnant patients with acute abdominal symptoms.

## Introduction

Pneumoperitoneum is a notable radiological finding that primarily indicates the presence of free air within the peritoneal cavity, often associated with significant intra-abdominal pathology [1]. In fact, over 90% of cases of pneumoperitoneum are attributed to the perforation of a hollow viscus, such as the stomach or intestines, which typically necessitates urgent surgical intervention. While this scenario is relatively common, a less frequently encountered variant is “spontaneous pneumoperitoneum”. This condition can arise from various atypical sources, including intra-thoracic issues, gynecological conditions, or iatrogenic factors resulting from medical procedures [2].

Physiologically, pregnancy induces significant changes, such as increased abdominal pressure from the enlarging uterus and hormonal alterations that affect gastrointestinal motility. These factors can complicate the presentation of abdominal conditions, making it difficult to distinguish between normal pregnancy-related symptoms and those indicative of serious pathology [3].

When faced with an acute abdomen in pregnant patients, clinicians often navigate the dilemma of avoiding CT imaging due to the associated radiation risks to the fetus. Alternative imaging modalities, such as ultrasound, may be preferred, yet they also present limitations in sensitivity and specificity [4].

Pneumoperitoneum itself is a rare occurrence during pregnancy, and instances of spontaneous idiopathic pneumoperitoneum, where all potential causes have been ruled out, are even more uncommon [5-11]. This report emphasizes the need for heightened awareness and consideration of this rare phenomenon, as it may influence current clinical practice by encouraging further investigation and discussion regarding appropriate diagnostic approaches in this vulnerable population. Understanding these dynamics is crucial for improving patient outcomes and guiding management strategies in similar cases. 

## Case presentation

The patient is a 41-year-old female who is 24 weeks pregnant. Her medical history includes a previous ectopic pregnancy and a diagnosis of substance use disorder. She was brought to the hospital by emergency medical services (EMS) after being found unresponsive, likely due to an overdose. EMS found the patient on the bathroom floor, unresponsive. In the field, she underwent two rounds of cardiopulmonary resuscitation (CPR) without the administration of epinephrine or defibrillation. However, she did receive a dose of Narcan, to which she responded appropriately, regaining some level of consciousness. Upon arrival at the hospital, she was intermittently responsive to questions but remained very lethargic.

Upon initial presentation, her vital signs were recorded as follows: temperature 96.3°F, heart rate 77, blood pressure 116/82, and respiratory rate 38, although she was saturating at 98% on room air. The elevated respiratory rate prompted the administration of 2 liters of oxygen via nasal cannula. In the emergency department, she remained hemodynamically stable. A physical examination indicated that her abdomen was taut, diffusely distended beyond the normal size of a gravid uterus, and diffusely tender. Due to a lack of prehospital notes, it is unclear if these physical exam findings were present on initial assessment in the prehospital setting or if this distension had developed during her prehospital intervention and transport. The rest of the physical examination was largely unremarkable.

**Figure 1:**
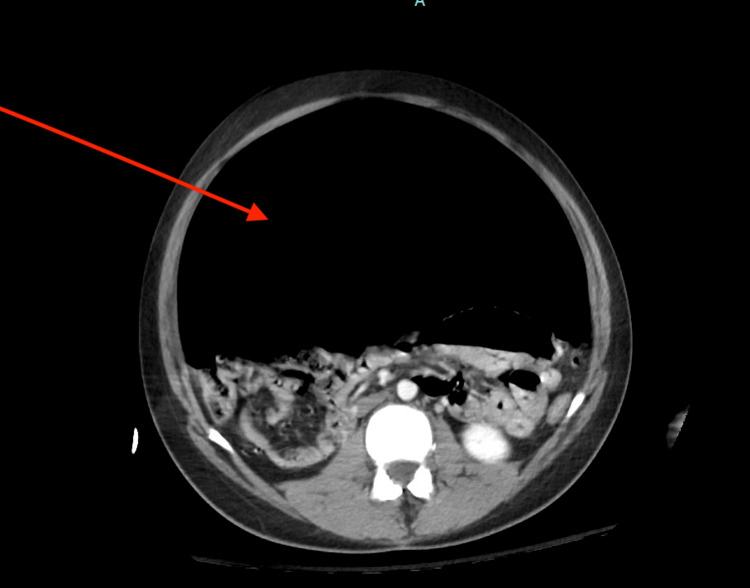
Axial View CT scan of the Patient With Intravenous Contrast Prior to Laparotomy Indicating Significant Free Air in the Abdominal Cavity, Highlighted by Red Arrow

**Figure 2:**
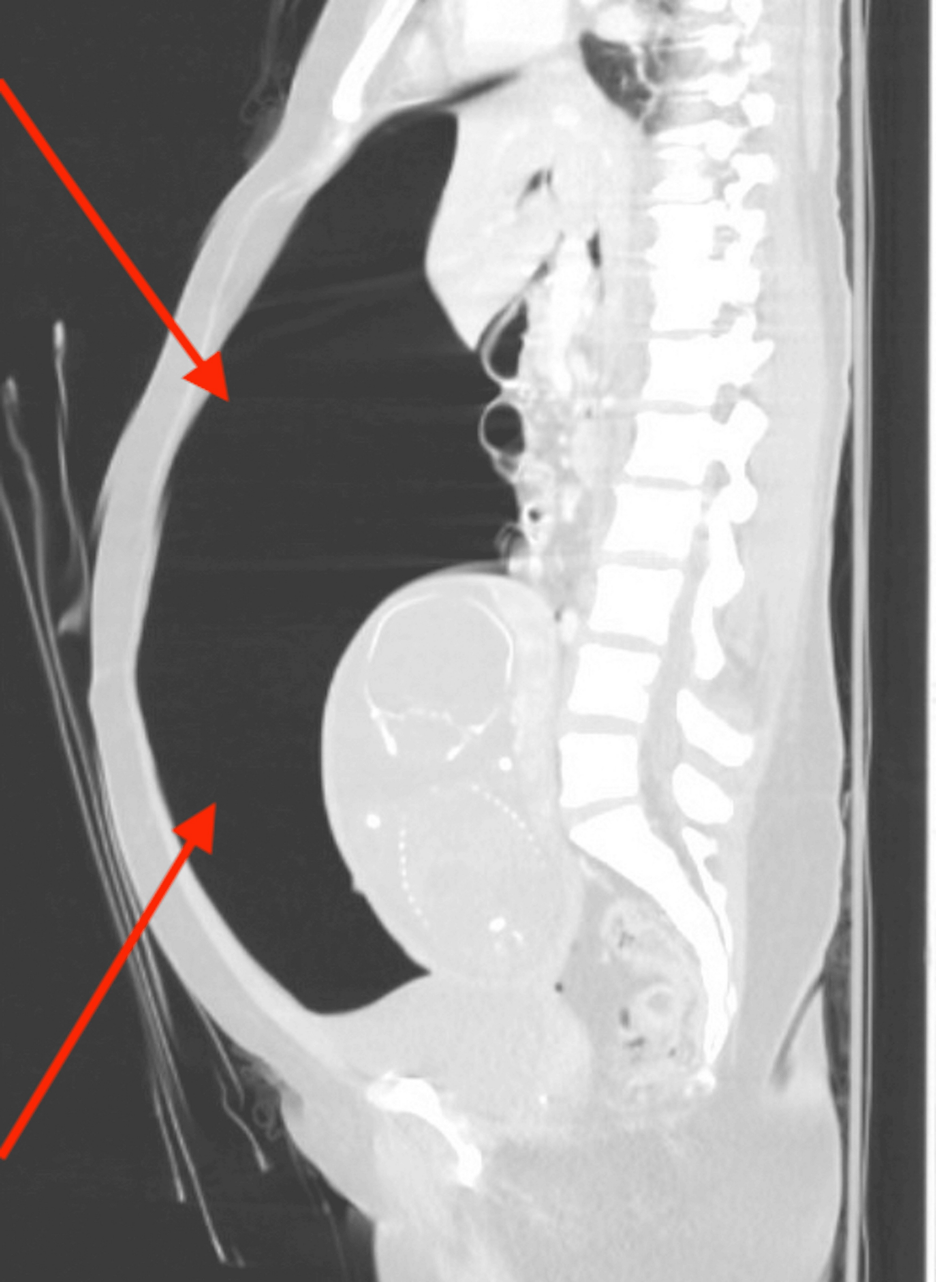
Sagittal View CT scan With Intravenous Contrast of Patient Prior to Laparotomy (Lung window to Further Highlight Significant Free Air in the Abdominal Cavity, Indicated by Two Arrows)

Laboratory tests revealed an elevated white blood cell count of 17,300 cells/µL, with neutrophils comprising 87.3% of the total. Lactic acid levels were measured at 1.5 mmol/L (Table 1). Additionally, urine toxicology results were positive for benzodiazepines, cocaine, and cannabinoids. In accordance with standard practice for evaluating intra-abdominal pathology in patients presenting with abdominal pain and distress, a CT scan was performed, revealing a significant amount of intra-abdominal free air without any evidence of free fluid (Figures 1, 2). 

**Table 1:** Preoperative Laboratory Values on Initial Presentation to the Emergency Department and Corresponding Reference Ranges

Test	Result	Reference
Hematocrit (%)	40.4	35-48
WBC (white blood count) x 10^3^/uL	17.3	4.5-11
Neutrophil (%)	87.3	50-70
Lymphocytes (%)	7.6	25-43
PLT (platelet count) x 10^3^/uL	356	140-450
Creatinine (mg/dL)	1.13	0.55-1.02
Lactic Acid (mmol/L)	1.5	0.5-<2

She was taken urgently to the operating room for exploratory laparotomy with concern for peritonitis. An upper midline incision from just below the Xiphoid process toward the umbilicus was created. This was above the fundus of the uterus. On entrance into the abdominal cavity, a large rush of free air was encountered. After completely opening the fascia, the abdominal contents were examined. No free fluid was identified. The entirety of the colon and appendix all appeared normal with no evidence of ischemia or perforation. The small bowel was run from the ligament of Treitz to the terminal ileum. There was no evidence of bowel pathology. The stomach was fully examined, revealing a small amount of air trapped at the lesser curvature, with no signs of frank perforation. The proximal duodenum appeared healthy, showing no exudate or evidence of perforation. To complete the exploration, the lesser sac was opened, allowing visualization of the duodenum after performing the Kocher maneuver, and access to the posterior wall of the stomach. There were no signs of free fluid or gastric contents. Air was instilled into the patient's stomach via nasogastric tube (NGT), and the stomach was submerged in saline solution, showing no air bubbles or leakage. A Jackson-Pratt (JP) drain was then placed in the left upper quadrant and lesser sac before the abdomen was closed. This drain was left in place for a prophylactic purpose due to inadvertent missed bowel injury.

Following the procedure, the patient remained sedated and intubated, and she was subsequently transferred to the Surgical Intensive Care Unit (SICU) in stable condition. She was extubated on postoperative day (POD) 1. Her course in the ICU was uneventful, leading to her being shifted to the medical/surgical floor on POD 3. During her hospital stay, she was evaluated by both the obstetrics/gynecology (OB/GYN) and gastroenterology (GI) teams, neither of which recommended any interventions.

On POD 3, the patient was placed on a clear liquid diet, with plans to conduct an upper gastrointestinal series if she did not tolerate it. She tolerated the diet well and was subsequently advanced to a regular diet without any complications. She ambulated around the unit without significant discomfort. Although there were plans for her discharge to an inpatient psychiatric facility due to concerns regarding substance use disorder and potential bipolar disorder, she declined this option and did not meet the criteria for involuntary admission. Upon discharge, the patient was notified of a follow-up appointment scheduled for two weeks later. However, despite multiple attempts to contact her, she has not attended several scheduled appointments with surgery, obstetrics/gynecology or maternal-fetal medicine.

## Discussion

In this case report, we examined a 41-year-old pregnant woman who presented with signs of pneumoperitoneum. Imaging revealed a significant amount of intra-abdominal free air, but surgical exploration showed no evidence of perforation. This raises questions about the potential causes of pneumoperitoneum in a pregnant patient, particularly considering her complex medical history and acute presentation.

One significant consideration during pregnancy is the risk of trauma, whether from accidental falls or domestic abuse, which can lead to visceral injury or perforation. The physiological changes during pregnancy, such as increased intra-abdominal pressure and alterations in the gastrointestinal tract, may also contribute to the development of pneumoperitoneum in atypical situations. The expanding uterus exerts pressure on surrounding abdominal organs, which can impair their function and increase the risk of perforation. Compression of the inferior vena cava can impede venous return, raising pressure in the venous system of abdominal organs and making tissues more fragile [12].

In reviewing the literature, while pneumoperitoneum is typically caused by visceral perforation in 85 to 95% of cases, 5-15% occur without such injury [13]. Notably, cases that underwent surgical exploration often reveal alternative causes. For instance, one study reported a case involving a sigmoid volvulus during pregnancy, where the patient presented at 32 weeks gestation with acute abdominal pain and signs of peritonitis. An ultrasound showed a fluid collection in the pelvis alongside massive pneumoperitoneum. This patient was immediately taken to the operating room, underwent exploratory laparotomy, and was found to have a perforated sigmoid volvulus. The procedure involved resection of the nonviable portion of the colon and a Hartmann’s procedure [14]. This illustrates that operative intervention may be necessary for patients presenting with massive pneumoperitoneum. In comparison to our case, these reports underscore the critical importance of diagnosing pneumoperitoneum in the context of pregnancy and the need for prompt surgical exploration to rule out serious underlying conditions.

Other conditions have been proposed as causes of pneumoperitoneum without hollow viscus injury. One such example is gas insufflation during sexual activity [15]; this, however, remains speculative in our case as we do not have definitive evidence linking this to our patient’s condition. Additionally, CPR can lead to pneumoperitoneum through barotrauma and mechanical forces applied during resuscitation efforts, resulting in air tracking from the thoracic cavity into the peritoneal space [16]. Literature supports surgical exploration in settings of peritonitis, as seen in our patient, or tension pneumoperitoneum, highlighting the need for careful evaluation in these scenarios [17].

Another relevant condition is pneumatosis cystoides intestinalis (PCI), characterized by gas-filled cysts within the intestinal walls. While PCI has been associated with other diseases, many cases occur independently and can lead to non-surgical pneumoperitoneum [13].

This case presents a unique instance of pneumoperitoneum without identifiable hollow viscus injury, suggesting a benign cause potentially linked to mechanical events or transient pressure changes within the abdominal cavity. It remains plausible that air entered the peritoneal space through alternative routes, although definitive conclusions cannot be drawn without further investigation.

In summary, this case underscores the complexity of diagnosing pneumoperitoneum in pregnant patients, particularly when surgical exploration reveals no perforation. Further investigation into similar reported cases could provide additional insights into the etiology of pneumoperitoneum and guide clinical management.

## Conclusions

The causes of pneumoperitoneum in pregnant patients present a diagnostic challenge, necessitating careful consideration of both common and atypical origins. In this case, we explored various potential causes, including the possibility that CPR may have introduced air from the thoracic cavity into the peritoneal space. Additionally, sexual activity could have contributed to the pneumoperitoneum by allowing air to move from the uterus. Other possibilities discussed include mechanical events or transient pressure changes within the abdominal cavity. This case underscores the importance of thorough evaluation and the necessity of surgical intervention when faced with acute abdominal symptoms and imaging suggestive of free air. Furthermore, ongoing coordination among surgical, obstetric, and psychiatric teams is essential for optimizing outcomes and addressing the complex needs of pregnant patients experiencing acute medical conditions.

## References

[1] Čečka F, Sotona O, Šubrt Z (2014). How to distinguish between surgical and non-surgical pneumoperitoneum?. Signa Vitae.

[2] Ramponi DR (2018). Pneumoperitoneum. Adv Emerg Nurs J.

[3] Cappell MS, Friedel D (2003). Abdominal pain during pregnancy. Gastroenterol Clin North Am.

[4] Yoon I, Slesinger TL (2025). Radiation exposure in pregnancy. StatPearls [Internet].

[5] Rathore YS, Aduri RS (2019). Idiopathic pneumoperitoneum presenting as acute abdomen. MOJ Clin Med Case Rep.

[6] Dhillon NK, Tatum JM, Ley EJ, Barmparas G (2018). Tension pneumoperitoneum after hanging. Am Surg.

[7] Bedard M, McInnis M, Banton K (2024). Spontaneous pneumoperitoneum presenting as an acute abdomen. J Surg Case Rep.

[8] Yamana I, Noritomi T, Takeno S (2015). Spontaneous pneumoperitoneum due to constipation. Case Rep Gastroenterol.

[9] McLaren O (2013). Spontaneous idiopathic recurrent pneumoperitoneum. J Surg Case Rep.

[10] Wang H, Batra V (2018). Massive pneumoperitoneum presenting as an incidental finding. Cureus.

[11] Sharma M, Ojha P, Taweesedt PT, Ratnani I, Surani S (2020). An intriguing case of pneumoperitoneum in a patient with COVID-19: do all pneumoperitoneum cases need surgery?. Cureus.

[12] Wasson E, Jones MJ, Fazili N, Burn P, Nagabushanam S, Vickery C, Ryan N (2021). Ischaemic bowel perforation secondary to a gravid uterus in a patient with treated inflammatory bowel disease and an ileoanal pouch: a case report. Ann R Coll Surg Engl.

[13] Mularski RA, Sippel JM, Osborne ML (2000). Pneumoperitoneum: a review of nonsurgical causes. Crit Care Med.

[14] Saghir MA, Fadhl HA, Mohammed S, Saeed A, Alsakkaf M (2024). A rare case report of the successful management of perforated sigmoid volvulus in a pregnant woman with massive pneumoperitoneum: first case in Yemen. Cureus.

[15] Jacobs VR, Mundhenke C, Maass N, Hilpert F, Jonat W (2000). Sexual activity as cause for non-surgical pneumoperitoneum. JSLS.

[16] Hargarten KM, Aprahamian C, Mateer J (1988). Pneumoperitoneum as a complication of cardiopulmonary resuscitation. Am J Emerg Med.

[17] Mani VR, Pradhan L, Gray S (2015). Development of pneumoperitoneum after CPR. Int J Surg Case Rep.

